# A Retrospective Comparison of the Performance of Two Negative Pressure Wound Therapy Systems in the Management of Wounds of Mixed Etiology

**DOI:** 10.1089/wound.2015.0679

**Published:** 2017-01-01

**Authors:** Theresa Hurd, Alan Rossington, Paul Trueman, Jennifer Smith

**Affiliations:** ^1^ Nursing Practice Solutions, Stevensville, Canada.; ^2^ Smith & Nephew, Hull, United Kingdom.

**Keywords:** negative pressure wound therapy, community care, silver dressings

## Abstract

**Objective:** Negative pressure wound therapy (NPWT) has been shown to be effective in the management of chronic and surgical wounds. The two most widely used modalities of NPWT are vacuum-assisted closure (V.A.C.) therapy (KCI, Inc., San Antonio, Texas) and the RENASYS NPWT system (Smith & Nephew, Hull, United Kingdom). This evaluation compares the performance of the two systems in the management of wounds of mixed etiology.

**Approach:** The evaluation is based on retrospective evaluation of more than 1,000 patients treated with NPWT in a community setting in Canada.

**Results:** Patients were well matched according to their baseline characteristics, including age, sex, and wound characteristics. No difference was seen between the two NPWT systems in terms of the percentage of patients reaching their predetermined treatment goal (90.0% and 93.6%, respectively). The time taken to achieve the treatment goal (median 8 weeks in both groups), percentage reduction in wound area (64.2% and 65.3%, respectively), and weekly rate of reduction in wound area (9.7% and 9.4%, respectively; *p* = 0.156).

**Innovation:** This evaluation is believed to comprise the largest cohort of patients treated with NPWT published to date and is one of the few studies that have attempted to provide a direct comparison of the performance of alternative NPWT systems.

**Conclusion:** Findings suggest that there are no clinically meaningful differences in the efficacy and performance of the two most widely used NPWT devices, based on consideration of a number of wound outcomes.

**Figure:**
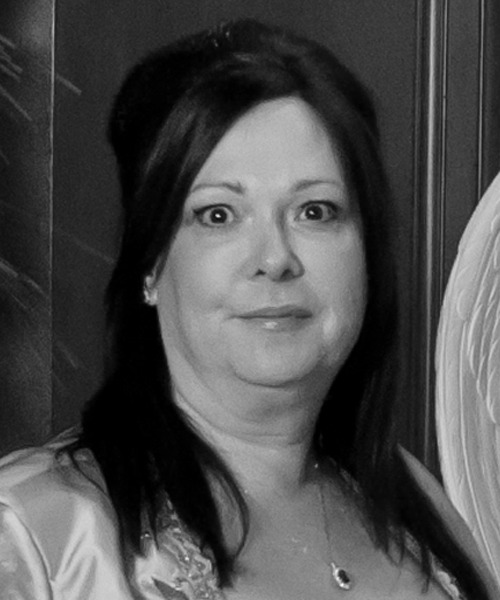
**Theresa Hurd, RN, MScN, PhD**

## Introduction

Negative pressure wound therapy (NPWT) is a well-established treatment option for the management of a range of chronic and acute wounds.^1^ NPWT is believed to contribute to wound healing through a number of mechanisms, including drainage of excess exudate, reduction of edema, and removing barriers to cell migration and proliferation, which when combined can accelerate the wound healing process.^2^

NPWT has been shown to have a positive impact on wound status in a number of different indications, including diabetic foot ulcers,^3^ venous leg ulcers,^4^ and burns.^5^ In addition to the clinical evidence, there is an emerging body of evidence to suggest that NPWT may be a cost-effective intervention when compared to advanced wound care, on the basis that it can increase the number of wounds healed and also reduce the time taken to heal wounds, in a number of settings and indications.^6–9^ As the body of evidence on NPWT has increased, a number of international guidelines and consensus statements have been produced to provide guidance on the appropriate use of this technology.^10–13^

The first commercial NPWT system (V.A.C.™; KCI, Inc., San Antonio, Texas) was approved by the U.S. Food and Drug Administration in 1995. Since then, a number of alternative commercial NPWT systems have been introduced into the market and both gauze and foam dressings have been developed for use with NPWT systems, providing healthcare professionals with a number of treatment choices. However, there have been relatively few attempts to compare the relative performance of NPWT systems. The two most widely used systems globally are vacuum-assisted closure (V.A.C.) therapy (KCI, Inc.) and the RENASYS™ NPWT systems (Smith & Nephew, Hull, United Kingdom). The two systems consist of a portable pump capable of delivering adjustable levels of negative pressure, linked by means of tubing to the wound filler. In both systems, the most commonly applied wound filler, which provides the interface between the pump and the wound, is a substantially equivalent black polyurethane foam. One difference is in the format of the “tubing” that links the wound filler and the pump. While the VAC system incorporates traditional tubing (TRACpad™), the RENASYS system includes a soft and compressible Softport™ device, designed to be comfortable for the patient. Both options are designed to transmit pressure to the wound bed and facilitate fluid removal. Rahmanian-Schwarz *et al.*^14^ conducted a head-to-head comparison of the two systems in the management of individuals with chronic and acute wounds before skin grafting. This study identified no difference in the healing rates, time on therapy, or complications between the two groups.

## Clinical Problem Addressed

There remains a notable absence of evidence on the comparative effectiveness of NPWT systems in practice across a range of wound types and clinical settings. The objective of this study was to compare the performance of the RENASYS NPWT system and V.A.C. therapy in the management of a range of chronic and acute wounds treated in a community setting in Canada.

## Materials and Methods

### Patients

This study describes a retrospective institution-wide audit of basic, anonymized data derived from patient records held by Nursing Practice Solutions, Inc., which co-ordinates the management of NPWT across two large community-based organizations and four acute care hospitals that serve a combined population of more than 3 million patients in Toronto, Canada. All patients with postsurgical wounds treated with NPWT between August 2009 and July 2012 were included in the analysis in accordance with the principles outlined in the Declaration of Helsinki. NPWT was either initiated in the acute care setting as part of the discharge plan or initiated in the community setting. In all cases, the patients were ultimately managed in the community. The application of NPWT was made according to an institution-wide protocol and the choice of device was typically made according to availability, not due to clinician preference. NPWT was excluded from patients with malignancy, eschar, or osteomyelitis in the wound, with uncontrolled diabetes and unexplored fistulae. To be eligible for NPWT, gold standard care must be in place (*e.g.*, compression bandaging in venous leg ulcer and off-loading in diabetic foot ulcer) to provide the best conditions for healing. The principles of Wound Bed Preparation^15^ were adhered to.

The initial treatment goal was recorded at the beginning of therapy and whether the treatment goal was met was a key outcome. Treatment goals included healing, readiness for surgical closure, management of bacteria, and management of wound exudate. Patients treated with NPWT were considered appropriate for NPWT according to local treatment protocols and were expected to adhere to best-practice principles on wound care, such as the use of compression for venous leg ulcers, off-loading, and blood glucose control for diabetic foot ulcers and general nutritional advice. The choice of NPWT system, including the decision over several treatment variables (foam or gauze dressings, and pressures settings), was determined locally by individual practitioners and the availability of NPWT devices. Local protocol and clinical judgment determined the use of any additional adjunctive therapies. For example, in wounds that had suspected localized bacteria burden or localized infection (*e.g.*, large amounts of drainage, odor), nanocrystalline silver (ACTICOAT Flex 3; Smith & Nephew) was applied according to local protocols. In wounds with large fluid volumes, after 2 weeks of therapy with continuous NPWT and once drainage was controlled, intermittent NPWT would then be applied for the remainder of the therapy. While there was no proactive attempt to match patients treated with each of the NPWT systems, patient characteristics were compared *post hoc* to determine whether there were any notable differences between the two groups.

Information on wound status was collected at baseline and weeks 3, 6, and 8 following treatment with NPWT. Performance criteria included wound area reduction at weeks 3, 6, and 8 and the proportion of patients meeting their predefined treatment goal. Additional information on adverse events, any NPWT device-related events, and adjunct wound treatments was also collected.

### Data handling and statistical analyses

Patients were excluded from the analysis if data were missing on any of the following baseline variables: age, sex, wound type, wound area at baseline, and treatment method. Differences between treatments in terms of binary variables, including proportion of achieving the treatment goal, were examined using a Fisher's exact test. Differences between treatments for percentage reduction in wound area were examined using unpaired *t*-tests. Kaplan–Meier analysis was used to estimate the median time to achieving a predefined treatment goal, and a log-rank test was used to test for a difference in the survival curve between treatments. All statistical analyses were conducted in SAS Version 9.1.3. Significance was set at *p*-value of 0.05 or below.

## Results

A total of 1,107 patients were analyzed, 808 of whom were treated with the RENASYS NPWT system (Smith & Nephew) and 299 of whom were treated with the V.A.C. system (KCI, Inc.). The two groups were well matched in terms of their demographics and baseline wound characteristics (Table 1). The majority of the wounds were postsurgical wounds that had developed complications. In particular, abdominal or colorectal wounds accounted for 23.3% versus 25.9% of wounds in the VAC versus RENASYS groups, respectively, followed by pilonidal sinus (8.7% vs. 11.1%) and tissue excision (6.8% vs. 7.2%). A range of other wound types included cardiothoracic, orthopedic, OBGYN, vascular, plastics (combined 14.1% vs. 14.7% respectively), and wounds originating from several other disciplines. There were no major differences in the origin of the wounds between the two groups.

**Table 1.** Patient demographics and wound characteristics

	*V.A.C.*	*RENASYS*
*N*	300	809
Age (years)	48.0	49.3
Male (%)	42.3	40.8
Wound duration (weeks) (min–max)	4 (1–34)	4 (0–260)
Wound area (cm^2^) (range)	18.8 (3–639)	18.8 (2–638)
Wound volume (cm^3^) (range)	56.6 (5–1578)	60.6 (7–1217)

V.A.C., vacuum-assisted closure.

**Figure 1.**
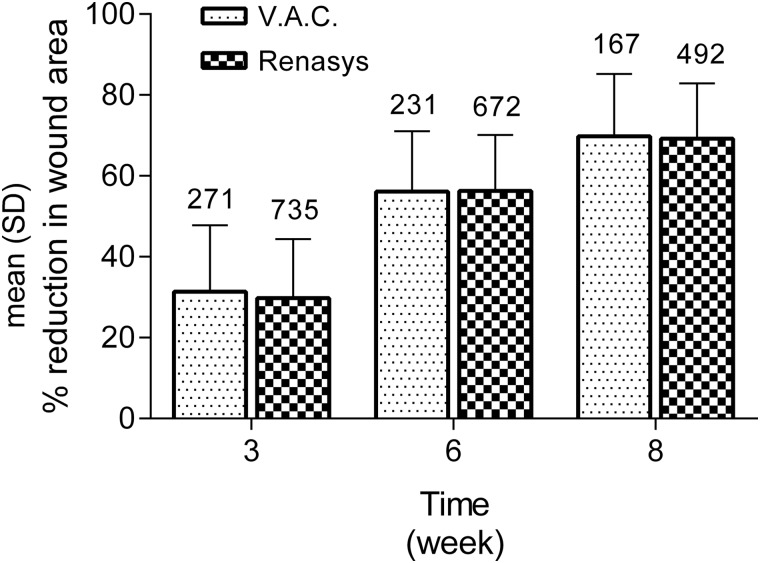
Median reduction in wound area at weeks 3, 6, and 8 in chronic surgical wounds treated with V.A.C.™ or RENASYS™. Mean ± SD and *N* number at each time point shown. No significant differences between NPWT devices were seen at any time point (*p* = 0.1364, *p* = 0.8524, and *p* = 0.6360 at 3, 6, and 8 weeks, respectively). NPWT, negative pressure wound therapy; V.A.C., vacuum-assisted closure.

Overall, 92.6% of patients treated with NPWT achieved their predetermined treatment goal (Table 2). No significant difference was seen between V.A.C. or RENASYS systems (90.0% and 93.6%, respectively). The time taken to achieve the predetermined treatment goal following the start of NPWT did not differ according to the NPWT device (median 8 weeks in both groups). The overall percentage reduction in wound area was also very similar between wounds treated with V.A.C. or RENASYS (64.2% and 65.3%, respectively, when NPWT was no longer required). This equated to a similar weekly rate of reduction in the wound area of 9.7% and 9.4% for V.A.C. and RENASYS, respectively, and there was no statistical evidence of a difference between treatments (*p* = 0.156). The reductions in the wound area over time are shown in Fig. 1 and illustrate a similar healing trajectory for wounds treated with either V.A.C. or RENASYS. No significant differences in wound area between NPWT devices were seen at any time point (*p* = 0.1364, *p* = 0.8524, and *p* = 0.6360 at 3, 6, and 8 weeks, respectively) (Fig. 1).

**Table 2.** Key clinical outcomes

	*V.A.C.*	*RENASYS*
Percentage of patients meeting their treatment goal	90.0	93.6
Median time in days to achieve treatment goal (range)	8 (1–20)	8 (1–35)
Mean % reduction in area throughout therapy	64.2	65.3
Median % reduction in wound area per week	9.7	9.4
*N*	299	808

V.A.C., vacuum-assisted closure.

### NPWT variables

Specific NPWT settings were not dictated by a strict clinical protocol but decisions were made according to clinical judgment. Some examples of variations in NPWT settings include reduced levels of negative pressure in patients experiencing pain and increased levels of negative pressure to improve fluid handling in highly exuding wounds. The majority of patients (91%) received between −120 and −150 mmHg, with a further 8% receiving −75 to 100 mmHg and only 1% receiving less than −75 mmHg. Ninety-four percent of patients received continuous NPWT and 6% were deemed to require intermittent therapy.

Of note is the high proportion of adjunctive silver dressings (ACTICOAT Flex 3; Smith & Nephew) used along with NPWT, with 34% of all patients receiving this combination therapy. These wounds were suspected of high bioburden or were considered at high risk of infection (*e.g.*, diabetic foot ulcers) and received nanocrystalline silver to reduce the risk of infection. Treatment goals in these patients typically related to reduction in signs of infection or reduction in bioburden. A subanalysis of these patients (Table 3) showed similar healing response when either RENASYS or V.A.C. systems were applied in combination with nanocrystalline silver dressings. More than 90% of the patients requiring this combination therapy achieved their treatment goal and had a similar weekly reduction in wound healing (63.9% and 68.2%, respectively).

**Table 3.** Use of silver as an adjunct to negative pressure wound therapy in wounds with suspected high bioburden or at risk of infection

*Measured parameter*	*RENASYS and Silver*	*V.A.C. and Silver*
Treatment goal achieved (% of patients)	94.8	92.0
Reduction in wound area (%) at end of therapy	63.9	68.2
Area of wound healed per week	9.5	9.4
*N*	291	88

V.A.C., vacuum-assisted closure.

## Discussion

Infection remains a major impediment to the successful management of complex wounds. There are several dressings or devices compatible with NPWT that may address the issue of infection. Local practice in this study dictated the use of nanocrystalline silver in wounds with signs of high bacterial burden in an attempt to improve their chance of wound progression. Even in these wounds, the combined use of NPWT (either device tested) along with adjunctive silver resulted in successful wound outcomes for a high percentage of patients, with more than 90% of patients achieving their treatment goal. The use of silver as an adjunct to NPWT in the hospital setting has been previously shown to significantly reduce the length of hospital stay, number of debridements, and overall length of treatment compared with NPWT alone.^16^ Furthermore, robust investigation will be needed to fully appreciate the impact of adjunctive use of silver along with NPWT in the community setting.

The study design has several limitations as well as some strengths. Some endpoints reported in this study are subjective, in particular, whether the predefined treatment goal was achieved. However, as consistent results between both subjective and objective (*e.g.*, wound dimensions) endpoints were observed, any doubt about the reliability of the subjective data can be allayed. While it is acknowledged that a direct comparison in a formal trial setting would be a more robust method of comparing the two technologies, it is also recognized that this would be a much more costly and lengthy approach. The more pragmatic approach adopted for this study generated a large sample of patients in naturalistic setting, and analysis of the patient cohorts showed that they were well matched in terms of age, sex, and wound characteristics and therefore suitable for meaningful comparison. A further advantage of the use of a naturalistic data set, as in this study, offers an indication of how therapies are used in real-world practice settings. It also allows for analysis and understanding of which variables to NPWT are used (and which ones are not used very much) outside the rigidity of a formal trial where these variables would have been dictated.

## Innovation

The majority of the published evidence on NPWT relates to the first commercially available system. The challenge for newer NPWT systems has been to demonstrate delivery of similar or identical wound care outcomes. The results of this study demonstrate that there are no clinically significant differences in outcomes that can be observed between the two different commercial NPWT systems. The choice of which system to use is then no longer dependent on clinical efficacy or the size of the body of evidence but can become dependent on other factors such as cost, availability, and personal choice.

## About the Authors

**Theresa Hurd, RN, MScN, PhD** is the president of Nursing Practice Solutions, Inc., Ontario, Canada, and has presented extensively on outcomes for patients with chronic wounds. **Alan Rossington, MEng, MSC** is a statistics manager employed by Smith & Nephew. **Paul Trueman, BA, MA** is the vice president of Health Economics and Outcomes Research at Smith & Nephew. **Jennifer Smith, MSc, PhD,** is a medical communications manager employed by Smith & Nephew with a focus on NPWT.

## Abbreviation and Acronym

NPWT: negative pressure wound therapy

## References

[1] Mouës CM, Heule F, Hovius SE A review of topical negative pressure therapy in wound healing: sufficient evidence? Am J Surg 2011;201:544–556 21421104 10.1016/j.amjsurg.2010.04.029

[2] Willy C ed. The Theory and Practice of Vacuum Therapy: Scientific Basis, Indications for Use, Case Reports, Practical Advice. Ulmfllonau, Germany: Lidqvist Book Publishing, 2006

[3] Armstrong DG, Lavery LA Diabetic Foot Study Consortium. Negative pressure wound therapy after partial diabetic foot amputation: a multicentre, randomised controlled trial. Lancet 2005;366:1704–1710 16291063 10.1016/S0140-6736(05)67695-7

[4] Vuerstaek JD, Vainas T, Wuite J, Nelemans P, Neumann MH, Veraart JC State-of-the-art treatment of chronic leg ulcers: a randomized controlled trial comparing vacuum-assisted closure (V.A.C.) with modern wound dressings. J Vasc Surg 2006;44:1029–1037 17000077 10.1016/j.jvs.2006.07.030

[5] Mouës CM, van den Bemd GJ, Heule F, Hovius SE Comparing conventional gauze therapy to vacuum-assisted closure wound therapy: a prospective randomised trial. J Plast Reconstr Aesthet Surg 2007;60:672–681 17485058 10.1016/j.bjps.2006.01.041

[6] Apelqvist J, Armstrong DG, Lavery LA, Boulton AJ Resource utilization and economic costs of care based on a randomized trial of vacuum-assisted closure therapy in the treatment of diabetic foot wounds. Am J Surg 2008;195:782–788 18355797 10.1016/j.amjsurg.2007.06.023

[7] Mouës CM, van den Bemd GJ, Meerding WJ, Hovius SE An economic evaluation of the use of TNP on full-thickness wounds. J Wound Care 2005;14:224–227 15909439 10.12968/jowc.2005.14.5.26776

[8] Whitehead SJ, Forest-Bendien VL, Richard JL, Halimi S, Van GH, Trueman P Economic evaluation of Vacuum Assisted Closure Therapy for the treatment of diabetic foot ulcers in France. Int Wound J 2011;8:22–32 20875048 10.1111/j.1742-481X.2010.00739.x PMC7950900

[9] Dowsett C, Davis L, Henderson V, Searle R The economic benefits of negative pressure wound therapy in community-based wound care in the NHS. Int Wound J 2012;9:544–552 22321132 10.1111/j.1742-481X.2011.00913.x PMC7950552

[10] Armstrong DG, Attinger CE, Boulton AJ, et al. Guidelines regarding negative wound therapy (NPWT) in the diabetic foot. Ostomy Wound Manage 2004;50(4B Suppl):3S–27S 15311482

[11] Birke-Sorensen H, Malmsjo M, Rome P, et al. Evidence-based recommendations for negative pressure wound therapy: treatment variables (pressure levels, wound filler and contact layer)—steps towards an international consensus. J Plast Reconstr Aesthet Surg 2011;64 Suppl:S1–S16 21868296 10.1016/j.bjps.2011.06.001

[12] Krug E, Berg L, Lee C, et al. Evidence-based recommendations for the use of Negative Pressure Wound Therapy in traumatic wounds and reconstructive surgery: steps towards an international consensus. Injury 2011;42 Suppl 1:S1–S12 10.1016/S0020-1383(11)00041-6 21316515

[13] Vig S, Dowsett C, Berg L, et al. Evidence-based recommendations for the use of negative pressure wound therapy in chronic wounds: steps towards an international consensus. J Tissue Viability 2011;20 Suppl 1:S1–S18 22119531 10.1016/j.jtv.2011.07.002

[14] Rahmanian-Schwarz A, Willkomm LM, Gonser P, Hirt B, Schaller HE A novel option in negative pressure wound therapy (NPWT) for chronic and acute wound care. Burns 2012;38:573–577 22100423 10.1016/j.burns.2011.10.010

[15] Schultz GS, Barillo DJ, Mozingo DW, et al. Wound bed preparation and a brief history of TIME. Int Wound J 2004;1:19–32 16722894 10.1111/j.1742-481x.2004.00008.x PMC7951422

[16] Siegel HJ, Herrera DF, Gay J Silver negative pressure dressing with vacuum-assisted closure of massive pelvic and extremity wounds. Clin Orthop Relat Res 2014;472:830–835 23813240 10.1007/s11999-013-3123-3 PMC3916586

